# To Our Patrons

**Published:** 1884-12

**Authors:** 


### TO OUR PATRONS.

Our publishers have been at great expense in bringing the Journal up to its present high standard, and up to this time have not troubled you with bills. But it is necessary now that they should collect up all outstanding bills in order to continue to give you a first class journal. We therefore ask each one of our subscribers who may receive a statement of their account to at once remit the amount.

The circulation of the Journal has increased rapidly since the present management have had control of it. It now has a circulation of over three thousand. With the support of the physicians in the South the circulation will be over doubled during the next year.

